# Before the Endless Forms: Embodied Model of Transition from Single Cells to Aggregates to Ecosystem Engineering

**DOI:** 10.1371/journal.pone.0059664

**Published:** 2013-04-15

**Authors:** Ricard V. Solé, Sergi Valverde

**Affiliations:** 1 ICREA-Complex Systems Lab, Universitat Pompeu Fabra, Barcelona, Spain; 2 Santa Fe Institute, Santa Fe, New Mexico, United States of America; 3 Institut de Biologia Evolutiva, UPF-CSIC, Barcelona, Spain; 4 European Centre for Living Technology, C Foscari University of Venice, Venice, Italy; Centre for Genomic Regulation (CRG), Universitat Pompeu Fabra, Spain

## Abstract

The emergence of complex multicellular systems and their associated developmental programs is one of the major problems of evolutionary biology. The advantages of cooperation over individuality seem well known but it is not clear yet how such increase of complexity emerged from unicellular life forms. Current multicellular systems display a complex cell-cell communication machinery, often tied to large-scale controls of body size or tissue homeostasis. Some unicellular life forms are simpler and involve groups of cells cooperating in a tissue-like fashion, as it occurs with biofilms. However, before true gene regulatory interactions were widespread and allowed for controlled changes in cell phenotypes, simple cellular colonies displaying adhesion and interacting with their environments were in place. In this context, models often ignore the physical embedding of evolving cells, thus leaving aside a key component. The potential for evolving pre-developmental patterns is a relevant issue: how far a colony of evolving cells can go? Here we study these pre-conditions for morphogenesis by using CHIMERA, a physically embodied computational model of evolving virtual organisms in a pre-Mendelian world. Starting from a population of identical, independent cells moving in a fluid, the system undergoes a series of changes, from spatial segregation, increased adhesion and the development of generalism. Eventually, a major transition occurs where a change in the flow of nutrients is triggered by a sub-population. This ecosystem engineering phenomenon leads to a subsequent separation of the ecological network into two well defined compartments. The relevance of these results for evodevo and its potential ecological triggers is discussed.

## Introduction

A key problem in evolutionary biology is the emergence of complex life forms under the cooperation of several interacting cells [1], [2]. Multicellularity emerged through evolution several times (at least 25) and has been a prerequisite for the generation of complex types of development [3]–[6]. This major transition brought division of labour and opened the door for the emergence of development and body plans [7]–[9]. But for many reasons, and in spite of its obvious importance, the evolution of multicellularity is not yet well understood. The fossil traces of the transition are still incomplete, although rapidly improving. However, dedicated efforts to unravel the phylogeny of multicellular living forms, the analysis of special model organisms and the cues provided by the presence of potential genetic toolkits predating the emergence of complex metazoans are defining the potential minimal requirements for the transition towards complex multicellular life forms.

This transition is particularly relevant for the critical changes that took place around 560 Myr ago, associated to the so called Cambrian event [1], [10] but its roots predate a much earlier time window, as indicated by the analysis of ancestral genomes. Moreover, the picture gets more complicated as we consider additional components related to the physical environment and the constraints and opportunities posed by ecological interactions. Actually, the multiple facets of the debate on the origins of multicellular organisms have to do with the role played by the different potential shapers of the event. These multiple factors are not independent, and are likely to have interacted in complex ways.

In general terms, the multicellular state is characterized by the existence of cell-cell interactions of some sort that provide a source for collective adaptation to energy limitations, physical fluctuations and eventually division of labor. In multicellular organisms, lower-level entities (cells) have relinquished their ability to reproduce as independent units and instead replicate exclusively as part of the larger whole. But long before a developmental body plan was even defined, in what has been dubbed the “pre-Mendelian world” [11], several layers of complexity were required. This as a particularly relevant problem deeply tied with the problem of hierarchies in evolution [12]–[13].

Before developmental programs allowed true multicellular organisms to emerge, single cells developed into monomorphic aggregates and later on into differentiated aggregates [13]. Moreover, cell adhesion mechanisms required for the emergence of multicellularity have a much early origin [14]. In this context, long before complex metazoans appeared, some key components of the toolkit were already in place. How did these components affected the transition to multicellularity is an open question, and theoretical models can help to address it.

Most mathematical and computational models dealing with early evolution of development assume that either genetic networks or even body plans are already in place or instead deal with pattern-forming colonies and their potential to form structures under given spatial and nutrient constraints [15]–[17]. However, less attention has been given to the physics associated to these processes, particularly in relation with early scenarios lacking fine-tuned genetic regulation of development. By physics we refer to two different levels. One includes diffusion, excitability, oscillations or even cellular interaction forces, which can be captured by cell sorting models based on energy minimization functionals [11], [18], [19], [20]. In this context, it is possible to evolve morphologies and observe the interplay between cell differentiation, growth and communication [21]–[23] with a properly defined optimization algorithm. The other level deals with the embedding of cells and organisms within a physical medium. Here, forces are closer to standard physics, i.e., the way cells might displace in the three-dimensional environment, how cells interact with the substrate and how cells find nutrients in a fluctuating medium. Previous work on physically-embedded artificial systems was pioneered by a number of researchers, who evolved artificial “organisms” [24], [25]. Related work has considered the interplay between genetic networks and morphogenesis [26]–[30].

In this paper we would like to address, under a well defined framework, some questions related to the pre-multicellular world where cell aggregates (but not true organisms or body plans) could develop, providing some key preconditions for multicellularity to emerge. In particular, we would like to understand how the physical context, cell-cell adhesion properties, ecological and epigenetic factors concur to favor the emergence of cell aggregates. Moreover, we would like to understand how the environment influences (and can be influenced by) the evolution of cell diversity and cell-cell interactions. These are, we believe, important pieces in the extended evolutionary synthesis provided by evo-devo [31].

We also try to see how this explicit three-dimensional embodiment can play a role in favoring the emergence of innovations. We explore such a pre-body plan scenario, and its potential for generating complexity, by allowing a physically-embedded model of a cellular community to freely evolve. The interaction between our simple evolved aggregates and the environment is shown to trigger the emergence of *ecosystem engineering* (EEN) an important component in macroevolutionary patterns [32]. EEN can be defined as a modification of the abiotic environment by a species that affects resource availability for other species [33]–[37]. Because it involves a persistent ecological modification, its presence implies the existence of ecological inheritance.

As we will shown below, a simple model of physically interacting cells with adhesion properties starting from a set of independent, genetically identical cells exploiting a single energy resource (from a given repertoire) evolves in time towards a spatially segregated community involving a trophic chain. The ecological network includes both a population of generalists feeding on all available food sources along with a population of specialized detritivores. The transition from the original monomorphic population to the spatially organized aggregate with ecological structure takes place through the emergence of an innovation grounded in evolving adhesion between cells and walls as well as cell-cell adhesion. In spite of its simplicity, it fairly well illustrates the potential of this type of model to explore the emergence of major transitions in pre-Mendelian scenarios.

### Environment, Cells and Physical Simulation

In our model, evolution takes place within a spatially confined environment (the spatial domain is a cube with floor and walls) where physical forces play a role as external constraints. Organisms are spatially embedded structures and their embodiment is relevant as it provides the proper link with the external world and the biotic scenario where other organisms inhabit.

**Figure 1:**
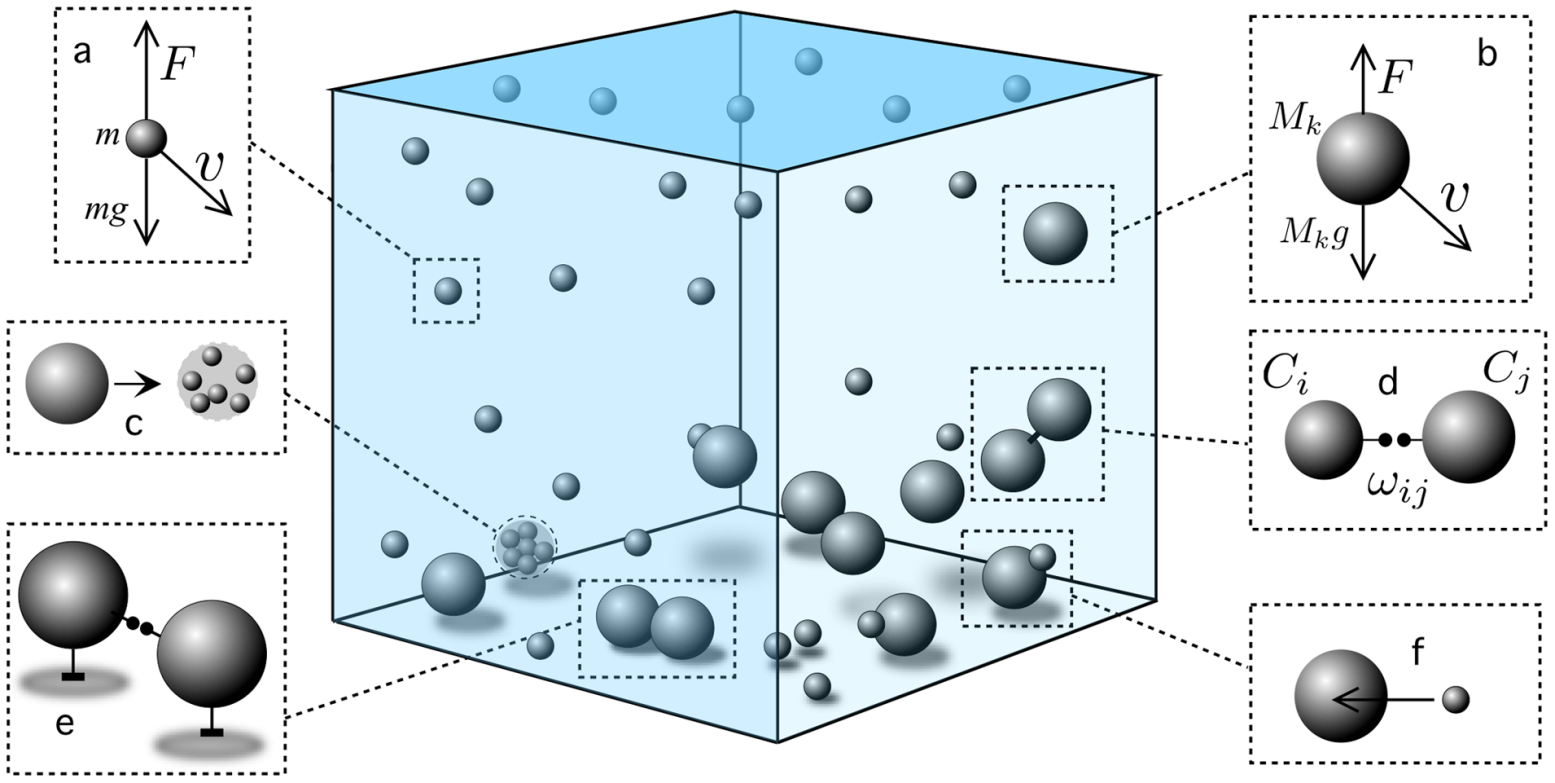
Basic scheme of the components of the CHIMERA model. The system is confined within a rigid cube with a floor where particles fall and to which cells can attach. Five additional square boundaries are also present which cannot be crossed. The upper boundary allows a flow of nutrient particles (here small spheres) at a constant rate. Particles fall under gravity (a,b) and experience local turbulence as a random velocity field. As they reach the floor, they can keep moving under the same flow and also disappear as they degrade (c) into detritus particles. Cells can evolve adhesion among them (d) as well as with the substrate (e). Finally, cells and particles (f) ineract through collisions. If the cell is able to exploit that particular type of energy, the particle involved disappears and is transformed into cell's biomass (see text).

Physical models of cell interactions have been developed for a broad range of problems involving multicellular assemblies [38]–[41] and our study follows some of the standard methods of computational physics [42]. Figure 1 displays a basic scheme of the system considered here along with the different components of the physical interactions that will be taken into account.

### 1.1 Cells and particles

Our starting point is a population of single-cell organisms, where each cell in the initial population is identical. Cells and particles are simulated with rigid bodies moving within a fluid-like environment. A cell (particle) has spherical geometry with radius 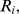 mass 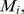 spatial position 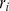 and velocity 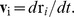 The motion of a cell is described by the standard second law:
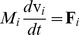
(1)


Numerical integration gives cell velocity at time 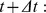


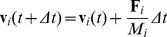
(2)where 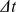 is the size of the integration step, and the total force acting on 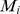 will be:



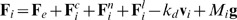
(3)applied to any cell is the sum of environmental forces 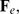 the gravitational field 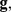 the collision force 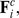 the cell-wall adhesion 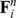 and the cell-cell adhesion 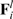 term.

### 1.2 Environmental forces

For simplicity, we asume that the external environment exerts the same force to every cell or particle, i. e.:
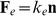
(4)where 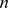 is a random vector with 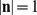 and 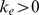 is a constant parameter that indicates the strength of the external field. This choice implies that external fluctuations in the fluid medium are homogeneous, thus affecting all parts of the system uniformly. This approximation thus neglects potential effects played by small-scale eddies, which might actually play a role in adaptational changes.

Movement of particles in a fluid is subject to dissipation, as defined by a viscous drag (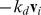) where 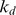 is the drag coefficient associated to the surrounding fluid. The effect of drag is to resist motion, making the particle gradually come to rest in the absence of other influences.

### 1.3 Cell-cell collisions

**Figure 2:**
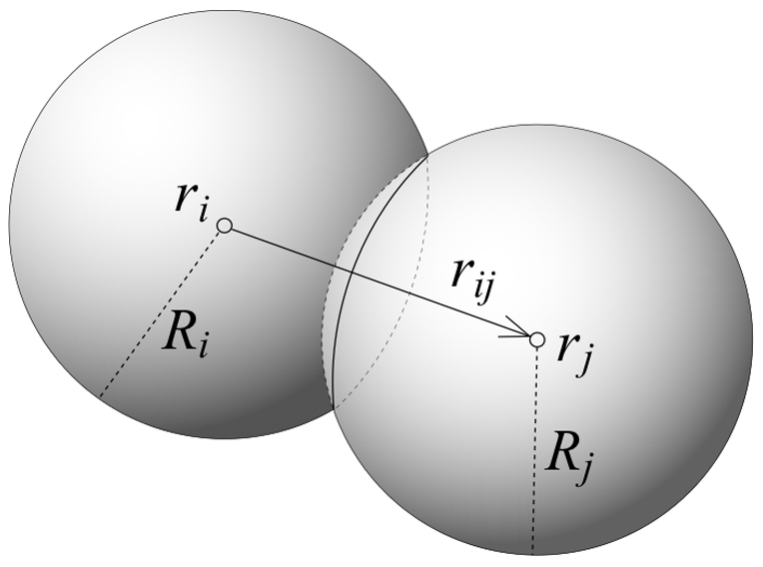
Simplified geometry of cell-cell collisions. Here, 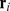 and 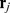 are the cell positions, 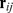 is the offset vector, 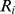 and 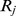 are the cell radius, and 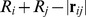 is the interpenetration depth.



We apply the discrete element method [43] to the computation of collision forces between the 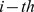 cell and all its interpenetrating cells, that is, cells located at a distance below a given threshold (see figure 2):
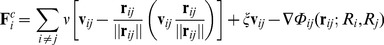
(5)where 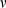 is the shear coefficient, 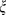 is the damping coefficient, 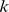 is the spring coefficient, and 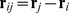 is the offset vector between the 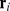 and 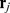 cell positions, and 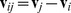 is the difference between cell velocities. The last term in the right-hand side gives the force resulting from a potential function associated to a soft-core interaction, namely.

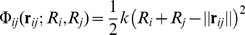
(6)Computation of spatial interaction forces requires 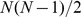 possible collision checks for 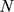 bodies in the worst-case scenario. In order to reduce this computational load we will use a spatial partitioning scheme. Here, a 3-D uniform grid subdivides the entire container volume in equally-sized square voxels [42]. All the bodies (cells and particles) are sorted by a mapping function 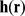 that computes their voxel index:
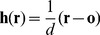
(7)where 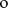 is the grid origin and 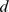 is the voxel size. For each body, we only check their collisions with different particles and cells belonging to a neighboring voxel, i.e., having indexes 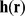 or 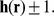 By adjusting the size of the voxel partition, we can minimize the total number of collision tests and enable the real-time simulation of large numbers of physically interacting bodies.

### 1.4 Cell-substrate adhesion

**Figure 3:**
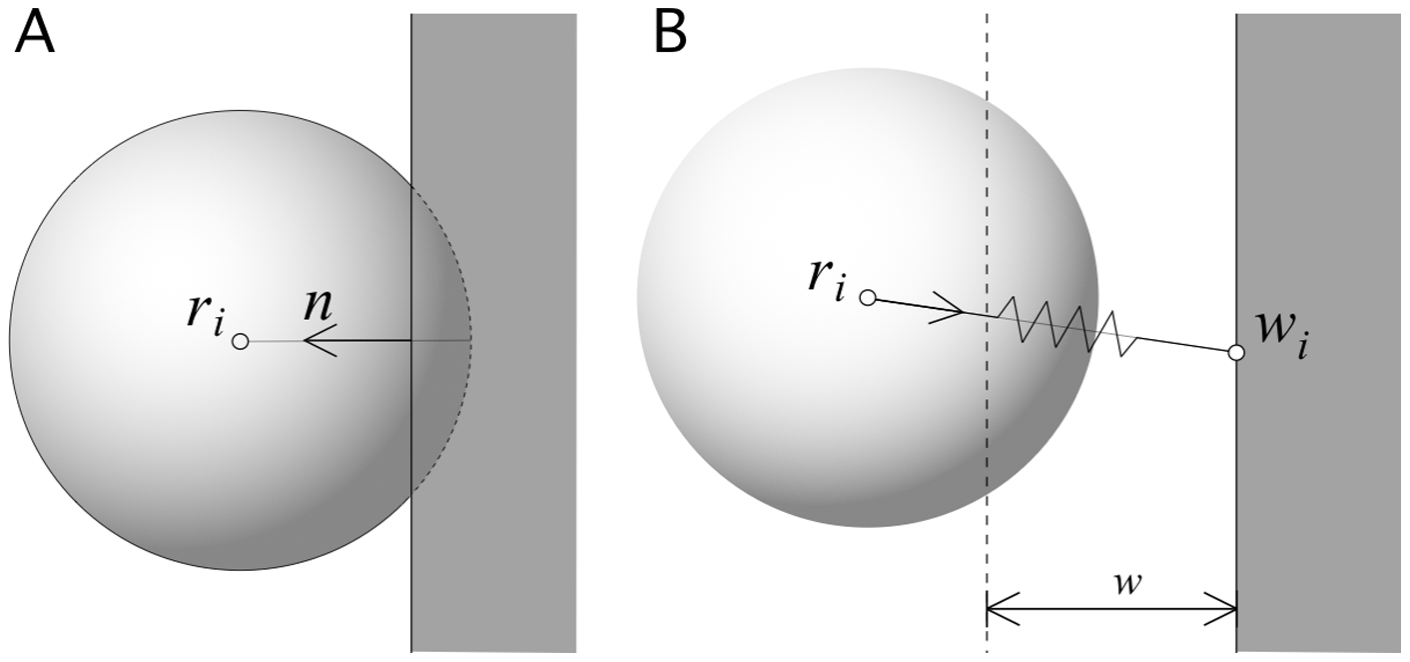
Possible interactions between cells and boundary walls. (A) Bouncing collision takes place when the cell interpenetrates the nearest wall any distance 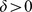 along the unit normal vector 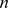 of the wall. (B) Cell-wall adhesion occurs when cell-wall distance is below some threshold 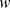 and according to adhesion probability (which depends on the cell genome, see text). In this case, a spring attaches the cell to a fixed point 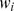 on the wall (see text).

Attachment of cells to surfaces may provide a favorable micro-environment (e.g., biofilms). Cell-wall distance 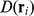 is:
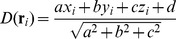
(8)where 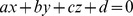 is the plane equation for the closest wall to the cell located at 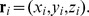 When a cell with adhesion strength to the substrate 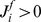 is closer than the adhesion range 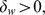 i.e., 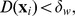 we attach a spring connecting the cell 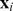 with its projection on the wall 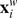 (see figure 3). Now, the wall spring exerts the following attraction force:
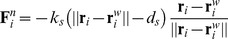
(9)where 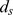 is the spring equilibrium distance, 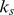 is the spring constant and 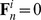 when the cell is not attached to any spring. Existing cell-wall springs can be removed with certain probability 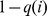 or when the spring length is above the maximal length, i.e., 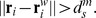 As we will see, cells can evolve cell-wall adhesion 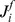 in order to maximize the intake of nutrient particles.

### 1.5 Cell-cell adhesion

Cells can form aggregates by attaching to other cells. Each cell has an intrinsic probability 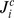 to create a new adhesion link. Given two close cells located at 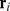 and 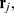 we will set an adhesion string connecting them with probability 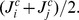 The adhesion force to any cell is the sum of forces contributed by all the active cell-cell adhesion springs:
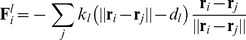
(10)where 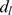 is the spring equilibrium distance, and 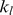 is the spring constant. Adhesion springs break spontaneously with rate 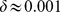 or when the spring is very large, i.e., 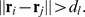


On the other hand, interpenetration collisions between cells and walls are not resolved with explicit forces (see figure 3A). When a body interpenetrates a wall, it bounces along its negative velocity direction, i.e., 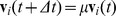 by a constant factor 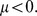 In addition, we relocate the body outside the wall (see below). This approximation ensures that particles will never move beyond container limits and does not change the main results presented here. The final cell (or particle) position is obtained from:
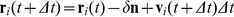
(11)where 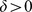 is the cell-wall interpenetration distance and 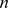 is the unit normal vector of the wall at the collision point with 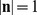 (see figure 3A).

Finally, the computation of particle force 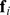 is more simple than in the case of cells. Food particles can collide with boundary walls, other particles and with cells (see below). But particles cannot adhere to the walls, or to any other body. The total force exerted on a particle is
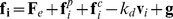
(12)where 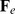 is the environmental force (as defined for cells, see above), 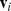 is the particle velocity and 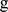 is the gravity field. Particle collision force 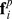 is similar to the equation used to compute cell collision responses (5) but using particle mass (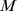) and radius 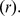 Similarly, the term 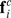 accounting for the collision response between any pair of interpenetrating particles mirrors equation (5).

We have calibrated the parameters associated to the physics as described above in order to avoid numerical instabilities. We have used small integration steps, i.e., 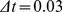 and fixed several physical parameters, including the cell and particle masses and the spring constants, to suitable values. An exhaustive exploration of the physical parameter space will be investigated elsewhere.

### Evolutionary Rules

Once our embodied, physical description of how interactions take place within our environment, we need to further extend our model by including evolutionary rules. Mechanical interactions, for example, can be understood in terms of predefined mechanisms (constrains derived from the nature of physical laws) together with sets of parameters that tune their relevance in terms of how different forces influence dynamics. In this paper we explore the impact of such parameter changes once the basic laws are already in place.

The introduction of physical constraints as part of the framework defining our cells and their interactions allows us to integrate different factors emerging at the boundaries between physics and biology. Moreover, the explicit nature of selection pressures associated with gathering nutrients from a spatially explicit, fluctuating environment allows to explicitly introduce ecological factors into play. In summary, the model takes into account physical embodiment, ecological and evolutionary constraints and allows interaction parameters among cells to change. Although no developmental programs or gene regulatory modules can emerge at this level, we will see how the degrees of freedom included in our approximation allow cell aggregates to emerge, along with innovation and the creation of niches.

At any given time, there is a set of cells 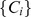 and a set of energy particles of identical mass 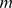 belonging to different energy types, here indicated as 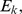 with 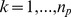 (here we use 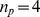 sources). Each cell 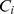 can in principle metabolize a subset of different sources with efficiency 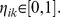 At the beginning, we start from a homogenous population of 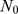 cells, each having mass 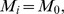 all feeding on the same resource, namely 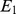 i.e., 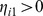 and 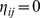 for all 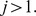 At every simulation step, we introduce 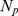 new particles in the system (here we use 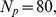 uniformly distributed over each class) starting at the top of our world. This number provides a quantitative value to the intake energy flow 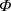 of particles entering the system.

Eventually, cells collide with nutrient particles and consume them according to the cell-specific efficiency 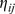 (a given nutrient particle is consumed only when this is non-zero, otherwise the particle is deflected and does not enter into the cell body). A particle of class 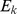 will be successfully grazed contributing to a linear mass increase:
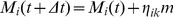
(13)


Once consumed, the particle is removed from the system. Nutrient particles have a characteristic lifetime and degrade at rate 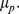 Cells consume a constant amount of energy 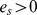 per time step to sustain themselves. A cell dies when mass 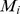 falls below a critical threshold 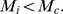 Cell death leads to disintegration and the release of 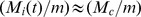 residual particles to the medium. These detritus particles will be consumed by detritivores when their associated efficiency is nonzero. Detritus particles have also a characteristic lifetime and degrade with a slower degradation rate, here fixed to 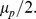


Cells divide once their total mass is at least twice their initial mass, 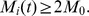 After reproduction, the mother cell 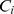 reduces its mass by 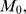 which is transferred to the daughter cell, say 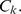 In other words, we have
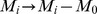


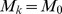



However, cell division is limited by the available surrounding space. Here, we allow the mother cell to reproduce only when its number of neighboring cells (i.e., cells within a radius of size 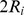) is below a given density threshold 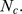 The new offspring is placed at a random location close to the mother cell, and specific rules ensure that this new cell is within the boundaries of the simulated medium.

The offspring cell 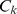 inherits metabolic efficiencies from the mother cell 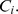 Metabolic efficiencies can be slightly changed, i. e.,
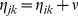
where 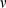 is non-zero with probability of mutation 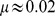 and zero otherwise. Here, the random 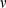 perturbation follows a Gaussian distribution with zero mean and standard deviation 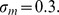 In addition, the offspring cell also inherits both cell-floor adhesion and cell-cell adhesion coefficients, respectively. Again, mutations are allowed to occur, and we have now:



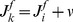
(14)

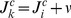
(15)


The values of these parameters are always normalized between zero and one. If a given mutation is accepted and the parameters are either lower than zero or higher than one, they are fixed to zero or one, respectively. Similarly, efficiencies are normalized so that the condition 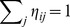 is always satisfied.

## Results

As a result of the previous set of rules and initial condition, we have a simple ecological food web involving a set of resources and a single specialist grazer, which takes 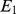 particles and grows at the expense of metabolizing them. Further degradation of particles leads to waste that is removed from the virtual tank and moreover cell death also generates an additional resource (the detritus compartment, 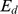). Ecologically, this is our starting point, which will evolve as organisms change their feeding preferences and as a consequence of interactions between organisms and their physical environment.

Given the potential for evolving physical parameters as well as nutrient intake-related parameters, our cells will be able to evolve within a range of possible adaptations. For example, given the number of potential food sources to be consumed, we will observe a spread from the original parameter set defined by
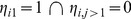
(16)to a continuum space of efficiencies



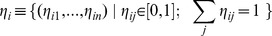
(17)As we will see below, these spread leads to a predictable outcome in a first phase of the evolution process, while it leads to an unexpected innovation later on.

### 1.1 Transition to generalism epoch

The first trend observed in all our simulations is a tendency from the starting specialization (all cells exploiting one source with maximal efficiency) to generalism: mutations allow to exploit other resources with less efficiency but overall this is a better strategy given the finite amount of energy particles. In our model, 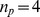 different types of energy particles are used. Additionally, since detritus particles resulting from cell death can also be consumed, a total of 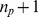 types of particles are available.

Nutrients enter the system from the upper layer at a constant rate 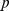 and degrade into waste at a rate 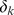. Under the absence of grazers, the time evolution of the number 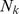 of energy particles of class 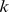 will follow a linear model 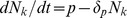 (for 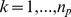) and thus each component will have an average steady value of 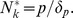 Then, the overall number of particles at the beginning (before grazing starts) will be 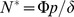 with 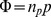 is the total flow of incoming particles. Right at the beginning, our grazers exploit only one source particle (say 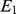). If no mutations were involved, we would observe a trend towards an equilibrium population of both cells and class-one particles over time.

The above describes the basic dynamics that occurs at the very early stages in our model, when only one source is being exploited. However, since we are interested in the long-term evolution, we would like to study the particle-grazer population dynamics. One of the first trends easily observed in our model is the tendency towards a broader range of exploitable resources, that is, grazers become generalists. Such trend towards a more opportunistic behavior occurs when the payoff given by the exploitation of a variety of resources outweighs the loss of efficiency as more resources are grazed.

The degree of generalism of a given individual is measured by means cell entropy 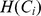 defined as as the normalized diversity of efficiencies:



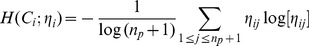
(18)where 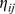 is the relative efficiency of the 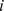-th cell when feeding on the 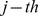 nutrient resource and 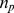 is the number of different resources. Notice that, in the initial configuration, all cells have entropy 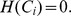 As defined, the degree of generalism will be zero when only one source is used (specialized diet) and will reach a maximum value for individuals grazing on multiple sources. Similarly, since the average efficiency for each cell is given by
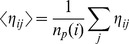
(19)where 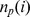 indicates the number of nutrient sources exploited by 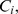 given the normalization of efficiencies we have simply 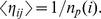


**Figure 4:**
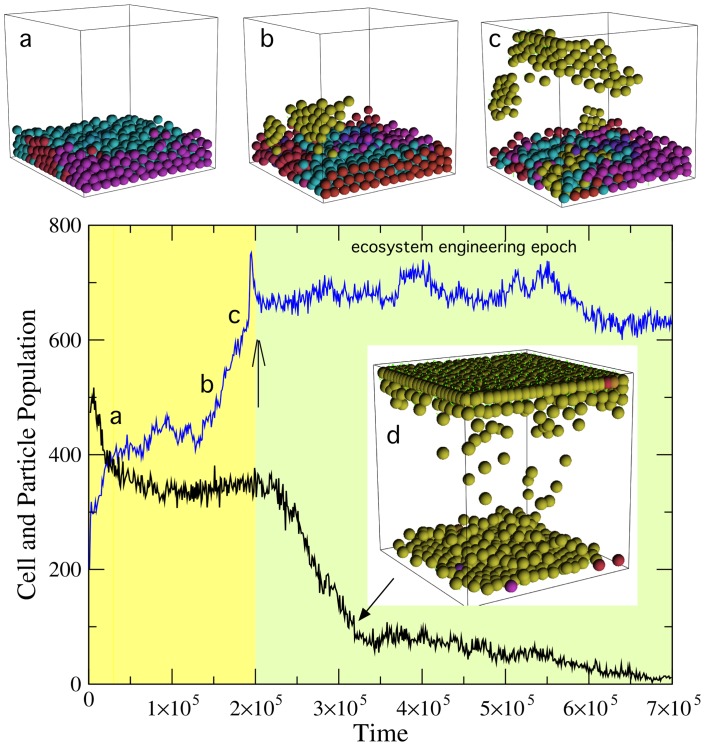
The time evolution of cells (blue) and particles (black) can be described with a sequence of processes. Before the transition to the inverted population state (inset) cells grow and divide as they also evolve their characteristic parameters. At the beginning, the cell population grows by adapting to the variety of energy sources and we can see a thick layer of cells (a) plotted at 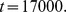 After a while, an increase in the surface displayed by the population increases its efficiency to gather particles. Such increase is reached by evolving cell-substrate and cell-cell adhesion. The state shown in (b) is found at 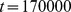 where we can appreciate a cluster of cells which appear to be climbing the surface of the cube. In (c) the cluster close to reaching the upper floor (here 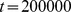) at this point the cell population does not experience further significant increase, but the number of particles decays to very low values. Here bright cells have high floor adhesion (where yellow indicates maximum adhesion) and darker colors correspond to cells with low adhesion or free-moving. Notice that adhesion evolves first in cells close to the wall boundaries. Here: 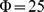 particles per timestep, 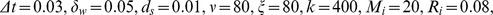 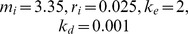 (see text).

The simulation shows a steady increase of cell-wall attachment and cell-cell adhesion followed by a steady increase in number of cells. This is a consequence of the fact that, in order to get access to falling particles, a larger surface is needed. Cells who attach to the surface can climb up the walls. Having a moderate cell-cell adhesion also helps in avoiding them to fall down. In this way, we have formed aggregates that are moving up as cells divide. The impact of this, along with the simultaneous tendency towards a generalist grazing behavior, can be seen in figure 4. The cell population (blue curve) grows rapidly as the aggregates start to emerge and expand, eventually covering the top layer.

During the process, groups of cells, often forming layers parallel to the floor, become increasingly larger. Three snapshots involving transient steps (a-c) are also displayed in figure 4, where we have used color-coded spheres to indicate the strength of the cell-wall adhesion. Lighter and spheres indicate higher and lower levels of adhesion, respectively.

### 1.2 Transition to ecosystem engineering epoch

**Figure 5:**
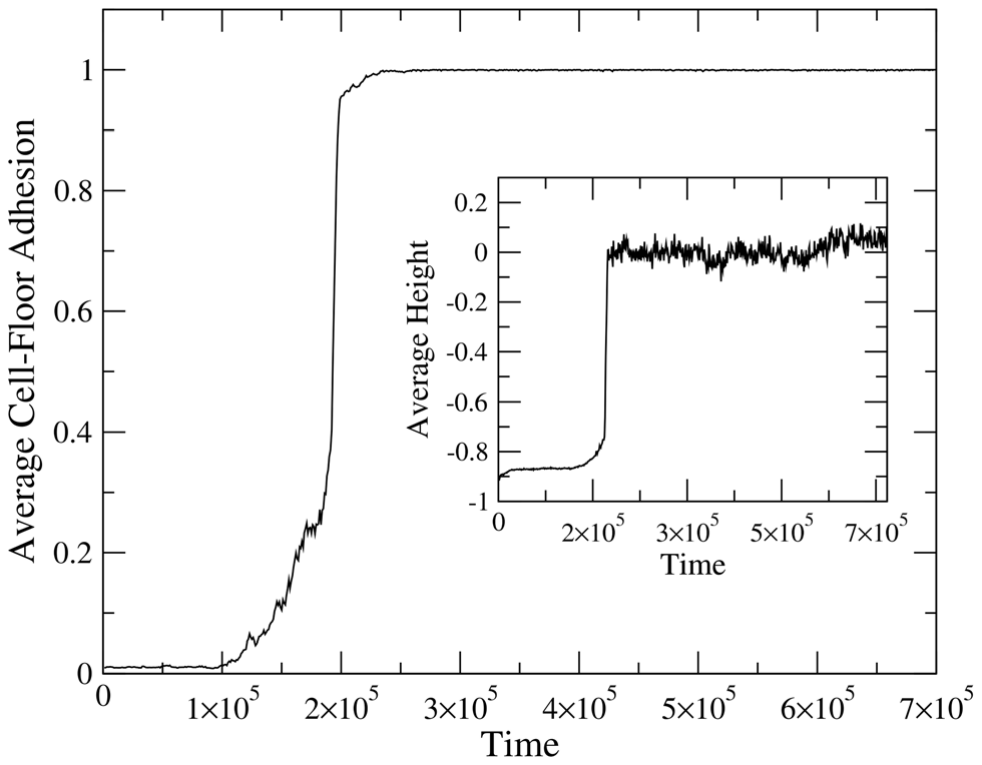
Sharp transition in the time evolution of average cell-floor adhesion. This transition is associated to the increasing height (inset) of the cell populations as further cells climb the walls towards the upper layer. Here the top floor is taken as the zero coordinate, whereas the bottom floor is taken as the 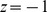 (normalized) minimum (see text).

As cells occupy the upper layer, the flow of nutrients further declines (filled arrow, figure 4) to reach very low levels. The kinetics of this process and how it is connected with evolving adhesion rates is summarized in figure 5, where we plot the average cell-floor adhesion (averaging over all the cell population) and the corresponding average height of cells within the cube (inset). Here we have used the top floor as the ground height (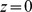) and the bottom one as the minimum. For convenience, we have normalized the later to 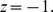 We can appreciate in these plots how rapidly the selection for higher adhesion occurs. As a consequence of this redistribution of cells through the upper part of the system, a whole redistribution of flows takes place, effectively triggering the emergence of a new ecological organization.

The most relevant result of our study is a key innovation made by our evolving cell populations as they “discover” that gathering particles is easier if they attach to the top floor of the system. As discussed above, cells approach the nutrient source by evolving cell-floor adhesion. This allows the population to expand its effective area for gathering nutrients, but it also favors more frequent interactions with particles, which are trapped between cells and the walls. This makes intake slightly higher than far from the boundary increases (and thus a higher fitness) because the sustained intake of nutrients. Eventually, cells with high floor adhesion (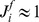) colonize the source of nutrients in a brief burst of super-exponential growth. This is illustrated by figure 4d, where we can see a snapshot of our system soon after cells have “discovered” the roof. In terms of the population dynamics, it is also observed that the number of free nutrient particles declines as they are more efficiently found and removed from the system.

**Figure 6:**
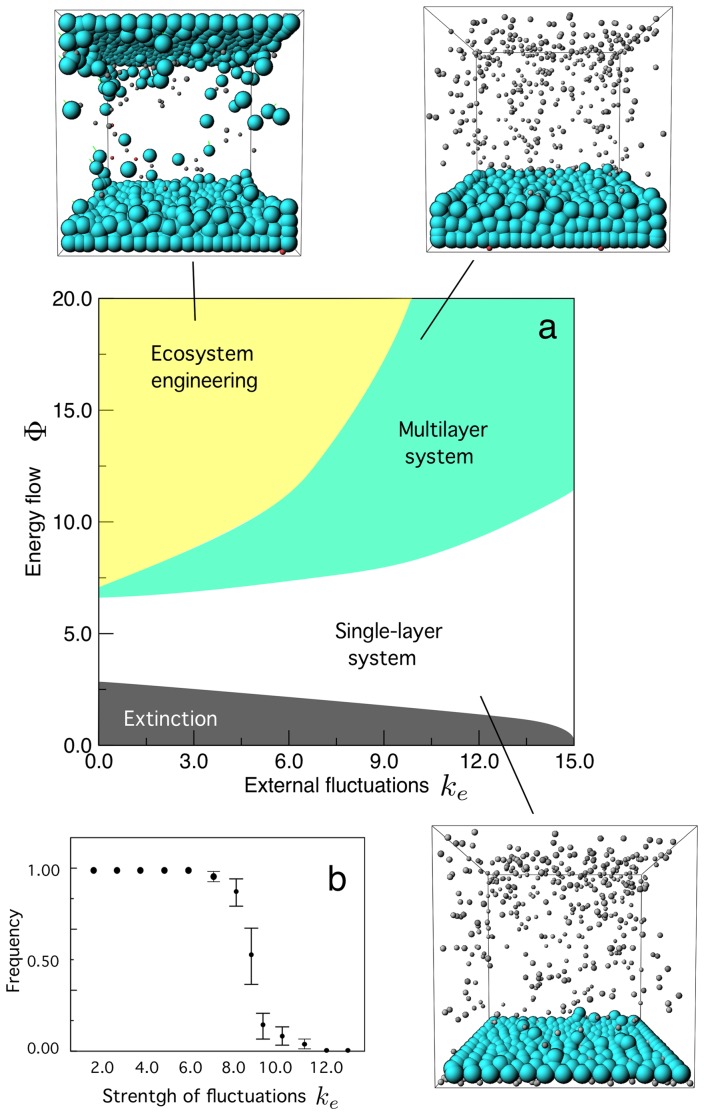
Interplay between energy intake and environmental fluctuations on the emergence of ecosystem engineering in CHIMERA's model. Here we have run CHIMERA using the same parameters of the previous figure but with different combinations of 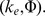 Four phases are found (a) including extinction (lower part), cellular monolayers as well as multilayered systems. Examples of each scenario are indicated with 3D snapshots where the adhesion strength between cells and the boundaries is color coded. A quantitative analysis of the transition from the EEN to the multilayer phase is indicated in (b), where 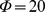 has been used along with different levels of fluctuation. Ten replicas of each parameter combination were used and 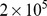 steps used to determine the final state. The standard deviation is also shown as error bars.

The emergence of EEN is common but requires both a high enough intake of energy (the flow of particles, 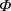) and a moderate level of environmental fluctuations. The analysis of these two parameters reveals several phases in the potential types of structures that the system generates. This is illustrated in figure 6 where we present a summary of our exploration of the parameter space defined by 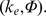 The choice of these two parameters is based on considering two relevant aspects associated to adaptation in evolving systems. The amplitude of the external fluctuations (as given by 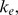 see equation 4) provides a measure of the random movements experienced by both cells and energy particles. In order to approximately determine the boundaries of these phases, the parameter space was partitioned into 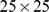 pairs and evolution experiments were done for each pair.

The phase diagram reveals four phases. As it should be expected, at low flow levels the initial population cannot sustain itself. However, it is interesting to notice that the domain of extinction gets smaller for higher levels of perturbation. This is understandable when we realize what that means. Since we use a closed system, both cells and particles are influenced by external noise and, at high levels, there is a coherent movement of both types of elements, which end to aggregate in the corners of the lower level. This introduces a natural increase of encounter rates and thus allows cell survival more feasible. In a way, the high environmental noise leads to a predictable outcome due to the presence of boundary conditions.

The next phase (indicated in white color) involves a more or less stable population of cells that are confined to the floor of the system. Adhesion is high for low 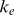 and decreases to small (but non-zero) levels at higher levels. Here the population presents the largest area compatible with the incoming flow. On top of this phase, several layers can be stabilized. Well-defined smooth multilayers are observed on the left part of the diagram whereas they become a bit rugged on the right side, due to the presence of noise. A moderate level of cell-cell interactions allows to stabilize the system. It is within this phase that the initially multilayered system can develop a more complex organization and EEN.

An example of the type of transition that occurs between phases is illustrated in figure 6b. Here we have fixed the incoming flow to 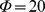 and determined the presence or absence of a top population of cells after 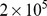 steps, using different 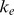 values. Averaging over ten replicas for each 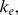 we obtain a phase transition close to the reported boundary. Around 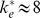 we observe a phase change separating two clear domains associated to the presence 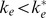 or absence 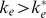 of a top layer. The error bars provide the standard deviation and are highest at the transition point, consistently with so called second-order transitions [44] where fluctuations strongly increase at criticality.

### 1.3 Emergence of detritivores

**Figure 7:**
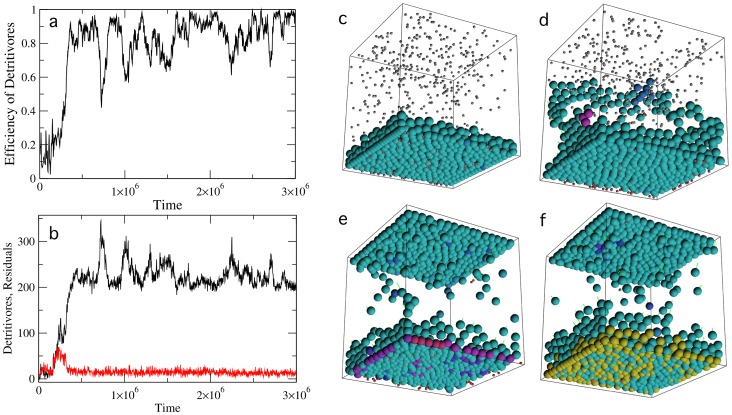
Dynamics of detritus grazing. In (a) the increase in efficiency rate of detritus-grazing cells is shown. In (b) we display the population dynamics of both detritivores (number of cells exhibiting some level of detritus feeding behavior) and detritus particles. In (c–f) four snapshots of the evolved system are shown. Detritivores evolve initially at the corners between wall boundaries thanks to the increased concentration of residuals. Afterwards, detritivores spread along the bottom plane and consume residuals produced by the disintegration of generalist cells coming from above. Four snapshots are shown in (c-f) at different times in the evolution of detritivores. Hot colors indicate the relative detritivore efficiency. Here: 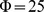 particles per timestep, 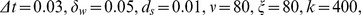 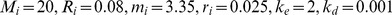 (see text).

A major consequence is derived from this innovation. As cells in the top layer start to dominate the whole flow of nutrients, something new happens. After the colonization is completed, cells die and their waste material falls to the bottom. The resulting detritus stays for a while and is eventually removed by degradation. But some cells have already developed a mild efficiency to exploit these particles. After falling from the roof, these cells will find themselves inhabiting a niche that is rich in a given type of energy source. After a while, they develop a higher efficiency and eventually become specialists. This is illustrated in figure 7a–b, where we can see that the efficiency of detritivores grows fast after the top floor population has been established.

In the inset of figure 7a we display an example of the stationary state This transition illustrates the nonlinear impact of ecosystem engineering [31]–[33]. By changing the flow of nutrients, the top floor population causes a physical change in the environment due to their spatial distribution. As it occurs with freshwater phytoplankton organisms, which intercept light by placing themselves on the top of the water column or with higher plants, producing litter form dead leaves, our ecosystem provides a clever example of this scenario. By modulating the flow of nutrients, which eventually shifts from nutrient-rich to detritus-dominated flows, an effective asymmetry is generated.

The process of niche creation and the emergence of ecosystem engineering promote a new feedback towards the way detritivores behave. If cell-cell adhesion is allowed to evolve, it can be shown that the detritivore compartment evolves significant adhesion among individual cells, which can be described as a new form of cell aggregate. It is interesting to notice that our aggregates do not cooperate in terms of sharing resources, but instead they emerge from selection constraints imposed by the requirement of an expanded area, which can only be obtained by attaching to the vertical surfaces. An additional advantage is obtained by living in higher locations: energy particles are falling near there and in fact, in the presence of fluctuations, it is likely that particles hitting the walls are captured by cells adhered to them. But the process has a discontinuity: by covering the roof, an accelerated transformation takes place, modifying the whole organization of the ecological assembly and allowing the emergence of an additional trophic compartment.

**Figure 8:**
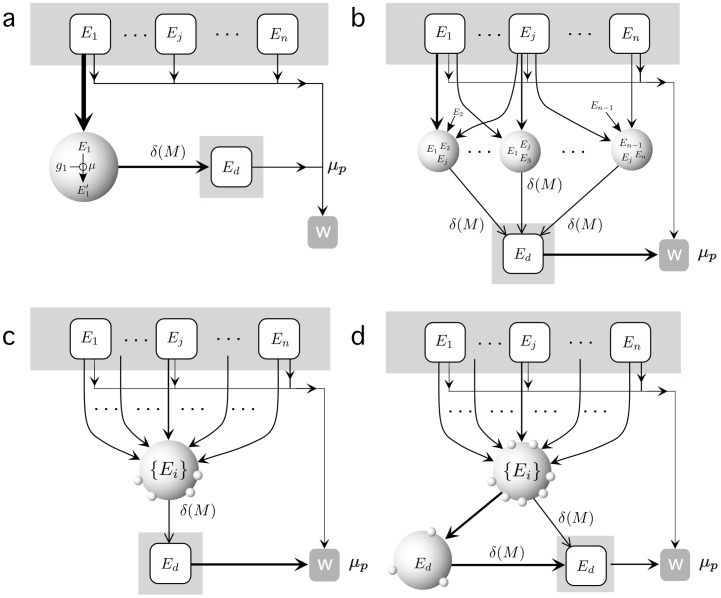
Chimera's food webs. (a) Flows in the initial food web, limited to a population of single-cell elements eating only one energy source (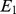) as defined by the CHIMERA model. The 1st nutrient is processed by the cells and transformed into cellular components high efficiency. At the end of the life cycle, the cell disintegrates into a number of residual particles. All molecules have similar degradation rates and get removed (right, lower box) from the system. After a while (b) an intermediate, diverse ecosystem is observed, with a large variety of cell exploiting different resources. This process proceeds until most cells become generalists, able to exploit all sources (c) whereas adhesion (indicated with smaller spheres) increases. Finally, once the population attached to the top floor has developed, another specialized population emerges at the bottom, fully composed by detritivores.

In figure 8 we summarize the ecological transitions experienced by our system as we cross through the different regimes. Here we have indicated the flows of matter from the external energy sources to waste. In fig. 8a, the initial state of our system is shown, with several sources of particles but only one being exploited by the single specialist. Such scenario is slowly replaced by a heterogeneous one (fig. 8b) marked by an increasing tendency to generalism: mutations affect efficiency rates and the potential for exploiting several resources. As evolution proceeds and cells develop adhesion (here indicated as small protrusions) along with a complete generalism (c). Here we indicate with 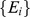 that cells use equally all types of nutrients with the same coupling. Eventually (d) the new niche of detritus-rich particles triggers the evolution of a specialized population of detritivores . This result is predictable, provided that the abundance of incoming particles is large enough.

## Discussion

In this paper we have presented a spatially-explicit model of evolution that incorporates ecology, physical embodiment and a very simple description of individuals. Our motivation was to provide an in silico platform to explore early stages of multicellular evolution under a pre-Mendelian scenario. Such pre-Mendelian world would be characterized by a higher morphological plasticity, far from the gene-regulated one of the Cambrian world 11,18. In order to explore the potential repertoire of multicellular patterns that can emerge under these conditions, we have used a specific setting where a vertical flow of nutrients is introduced at the upper boundary of our system.

If the initial steps towards life took place in water, an appropriate model considering the role of physical interactions must take into account (a) events taking place within a fluid and (b) those associated to physical exchanges between individuals and individuals with the boundaries of the system (such as the sea bottom or a pond floor). By considering these basic forces, we introduce a minimal set of (possible) rules of interaction that can evolve through time. Such rules allow our artificial cells to explore their environment, interact and make decisions. But it also provides a very basic framework to explore the potential for finding patterns of pre-developmental pathways based on interactions among single-celled entities.

Despite the simplified nature of our simulation approach, which prevents (in particular) the emergence of complex regulatory networks and introduce limitations to the spatial organization of cell aggregates, several nontrivial results emerge. The evolutionary transition from the floor to the top layer leads to ecosystem engineering [27] and, as far as we know, this is the first example of such event happening in an artificial life system. By changing the actual flow of nutrients, they cause a physical change in the environment due to their physical distribution. Moreover, by doing so they also allow the emergence of a specialized, spatially segregated compartment of detritivores. Following the classification scheme from [35], our artificial creatures are autogenic engineers: they change their environment mainly via their own physical structures. Here the main structure created is a cell barrier associated to cell-floor interactions. The tempo of the transition exhibits a marked acceleration as the cell aggregates approach the top of the world. We can actually see that the cell population moves through its world by increasing in size until they detect the asymmetry associated to the top-down and start exploiting it. As it occurs with any other system out of equilibrium, our system is sustained by a gradient linked to a given flow of energy. In terms of the innovation process, the punctuated nature of the change is due to the transient time required in order to physically reach the energy-rich boundary and exploit it.

Future work should consider the explicit introduction of minimal genetic networks, the potential for cooperation in terms of nutrient sharing, an active role of our organisms in modifying their environmental conditions (thus adding an additional layer to the potential for ecosystem engineering) and variability of spatial conditions. These in silico experiments could be compared in some cases with evolutionary sequences of growth and selection observed in experiments involving transitions from single-cell to multicellular aggregates [45]–[49]. In this context, it can help designing and interpreting experimental approaches based on the introduction of selection favoring given traits. All these extensions of the CHIMERA model will allow us to approach relevant questions concerning the tempo and mode of the transition to multicellularity and how the different players (ecology, genetic interactions, physical embodiment and path dependence) influence the final outcome.

Finally, CHIMERA can be used to explore the effect of external events involving large extinctions and their aftermath. Recovery from mass extinction provides a unique insight into how communities are rebuild and the role played by different biotic and abiotic components in the reconstruction of a paleocommunity [50], [51]. This is very well illustrated by the end-Permian extinction event [52], [53] which devastated most species, to the point of near annihilation of complex life forms. The existing fossil record of the process and the patterns of change is very rich and well established [54] an illustrates the power of looking at macroevolutionary dynamics using multiple perspectives, from geological data to multispecies interactions. Some theoretical and computer models [55]–[57] have shown that a systems approach to these events can help to determine potential causal scenarios of recovery. But these models lack the physical embodiment that is characteristic of our model both at the individual and ecosystem levels. Since we also can trace the patterns of change in sediment particles and have a well-established segregation between trophic levels, we have a unique way of evaluating the relative contributions of ecological processes of competition or cooperation as well as the underlying evolutionary changes taking place at the individual level. These in silico experiments can help understanding the role played by altruism and cooperation and how they emerge and the nature of the resulting cooperative units [58], [59].
